# Positive epistasis among ribosomal mutations drives high-level streptomycin resistance in *Escherichia coli*

**DOI:** 10.1128/aem.00377-26

**Published:** 2026-06-18

**Authors:** Fengjun Xu, Yue Xing, Yujie Men

**Affiliations:** 1 Department of Chemical and Environmental Engineering, University of California, Riverside 684488, Riverside, California, USA; Michigan State University, East Lansing, Michigan, USA

**Keywords:** positive epistasis, *rpsE*, *rsmG*, *rpsL*, streptomycin, antibiotic resistance

## Abstract

The widespread use of antibiotics has raised serious concerns about the emergence and dissemination of antibiotic resistance in the environment. Studies reveal that environmental exposures to sub-inhibitory level antibiotics and other low-level non-antibiotic pollutants can promote the development of high-level antibiotic resistance via *de novo* mutations. However, specific genotypes conferring strong resistance in resistant mutants, particularly the roles of co-occurring mutations, remained poorly understood. This study fills this knowledge gap by constructing site-specific *Escherichia coli* mutants and demonstrating that positive epistasis among ribosomal mutations conferred the observed strong streptomycin resistance in evolved resistant *E. coli* isolates. Three ribosomal mutations (*rpsE^D142N^*, *rpsL^R86S^*, and *rsmG^W150fs^*), which co-evolved under environmentally relevant selection pressures, acted synergistically to confer resistance far exceeding that of each individual mutation. The interactions between the *rsmG* frameshift mutation and other reported *rpsL* point mutations were also investigated. These findings unravel the essential but previously overlooked role played by specific co-evolving mutations in the acquisition of both high-level streptomycin resistance and fitness compensation.

**IMPORTANCE:**

This study reports a strong positive epistatic interaction that evolves under the environmentally relevant conditions, where microbes are exposed to sub-inhibitory concentrations of antibiotics along with other low-level pesticides. It elucidates how such interaction confers high-level streptomycin resistance to bacteria. This phenomenon contributes to the fundamental understanding of antibiotic resistance mechanisms mediated by target-altering mutations in nature. Findings in this study also underscore a strong and increasing need to investigate whole genomes of antibiotic-resistant bacteria and identify potential epistatic mutations conferring resistance phenotypes. The integration of those genetic mutations as antibiotic resistance biomarkers will complement the resistome profiling and enable more accurate antibiotic resistance monitoring and environmental risk assessment.

## INTRODUCTION

The widespread use of antibiotics has led to the emergence and dissemination of antibiotic resistance in the environment, posing one of the greatest threats to the health of humans, animals, and the environment (1–3). To mitigate the increasing risk of antibiotic resistance and promote One Health, substantial efforts have been made to control antibiotic usage and monitor antibiotic resistance determinants (i.e., antibiotics, antibiotic resistance genes, and antibiotic-resistant bacteria) in various environments. High-throughput sequencing-based antibiotic resistome profiling has become a widely applied tool to evaluate antibiotic resistance risks in a given environment (4, 5). However, resistome-based monitoring may involve false positives (i.e., genotypes do not necessarily lead to resistance phenotypes), especially for antibiotic resistance mechanisms involving target-altering mutations that reduce the antibiotic binding affinity to the target enzyme (5). Besides, resistome profiling may also generate false negatives when resistance is mediated by chromosomal single-nucleotide polymorphisms (SNPs) that are not represented in antibiotic resistance gene databases. Thus, it is crucial to identify and experimentally validate specific target mutations conferring antibiotic resistance, which may serve as complementary biomarkers or be integrated into existing antibiotic resistance gene databases for the surveillance of antibiotic resistance.

Resistance to several classes of antibiotics may be attributed to target alterations, including aminoglycosides (inhibiting ribosomal proteins). Among them, streptomycin is a commonly used one to treat bacterial infections in humans and animals (6, 7). It irreversibly binds to the 30S ribosomal subunit and disrupts protein synthesis. Bacteria can develop resistance by mutating genes encoding the target enzyme (8, 9), and the effectiveness of streptomycin is thus reduced due to the weakened drug-ribosome interactions. High-level streptomycin resistance is most frequently associated with mutations in *rpsL*, which encodes the crucial ribosomal protein S12 in the 30S ribosomal subunit (10). Loss-of-function mutations at *rsmG* (also known as *gidB*), which encodes the ribosomal RNA small subunit methyltransferase G, have been detected separately or together with *rpsL* mutations, and individual *rsmG* mutations may cause mild streptomycin resistance (11, 12). Those mutations were not only observed under clinically relevant conditions but also under exposures to sub-inhibitory levels of antibiotics and environmental levels (below mg/L) of other non-antibiotic co-occurring pollutants (13–18). Our previous studies revealed that co-exposure to 1/5 of the minimum inhibitory concentration (1/5 MIC) and pesticides at environmentally relevant levels promoted the development of high-level streptomycin resistance in *Escherichia coli* populations, where both target mutations (e.g., *rpsL^R86S^* and *rsmG^W150fs^*) were detected at the same frequency (13, 19).

Although individual target-altering mutations have been identified, evidence linking specific genotypes to the resistance phenotype is limited because in many previous studies only mutations of target genes were examined using Sanger sequencing, whereas the whole-genome information was missing. Consequently, there is a knowledge gap regarding the interactions of co-evolved mutations in the genome and how they could contribute to resistance phenotypes. To date, there are only a couple of studies that incidentally touched those questions while aiming at other topics. One study showed that individual *rpsL* or *rsmG* mutations could act synergistically with the streptomycin-degrading gene (*strB*) on a plasmid, resulting in resistance levels much higher than those conferred by *strB* alone (20). However, the interactions between target-altering mutations at *rpsL* and *rsmG* in bacteria without resistant plasmids still remained unknown. In another study focusing on streptomycin production in *Streptomycetes* species, some results reflected a synergistic or additive effect on resistance in mutants, where both *rpsL* and *rsmG* mutations were detected by Sanger sequencing (21). However, since those mutants might harbor additional uncharacterized mutations not verified by whole-genome sequencing, no concrete conclusion on epistasis could be made. Therefore, a more specific and targeted investigation using appropriate molecular tools is in great need to interrogate potential epistasis among co-evolved mutations that may have been previously overlooked but can shape high-level antibiotic resistance profiles.

Many studies have highlighted the role of epistasis in shaping antimicrobial resistance evolution, including context-dependent interactions between resistance mutations (22–25). Here, we systematically investigated the interactions among ribosomal mutations in *rpsL* and *rsmG* previously reported in streptomycin-resistant *E. coli* isolates. Site-specific mutants carrying individual or combined ribosomal mutations were constructed by introducing desired mutations into the chromosome of a wild-type, streptomycin-susceptible *E. coli* strain. The resistance level of each constructed mutant was compared. A strong positive epistasis among specific ribosomal mutations on the development of high-level resistance was observed and corroborated by reversing the mutations to the wild-type allele in the native-resistant strain. The implications of those new findings for future antibiotic resistance studies, environmental monitoring, and risk assessment were further discussed.

## RESULTS AND DISCUSSION

### Strong positive epistasis among three ribosomal mutations conferred substantial increase in streptomycin resistance

**Fig 1:**
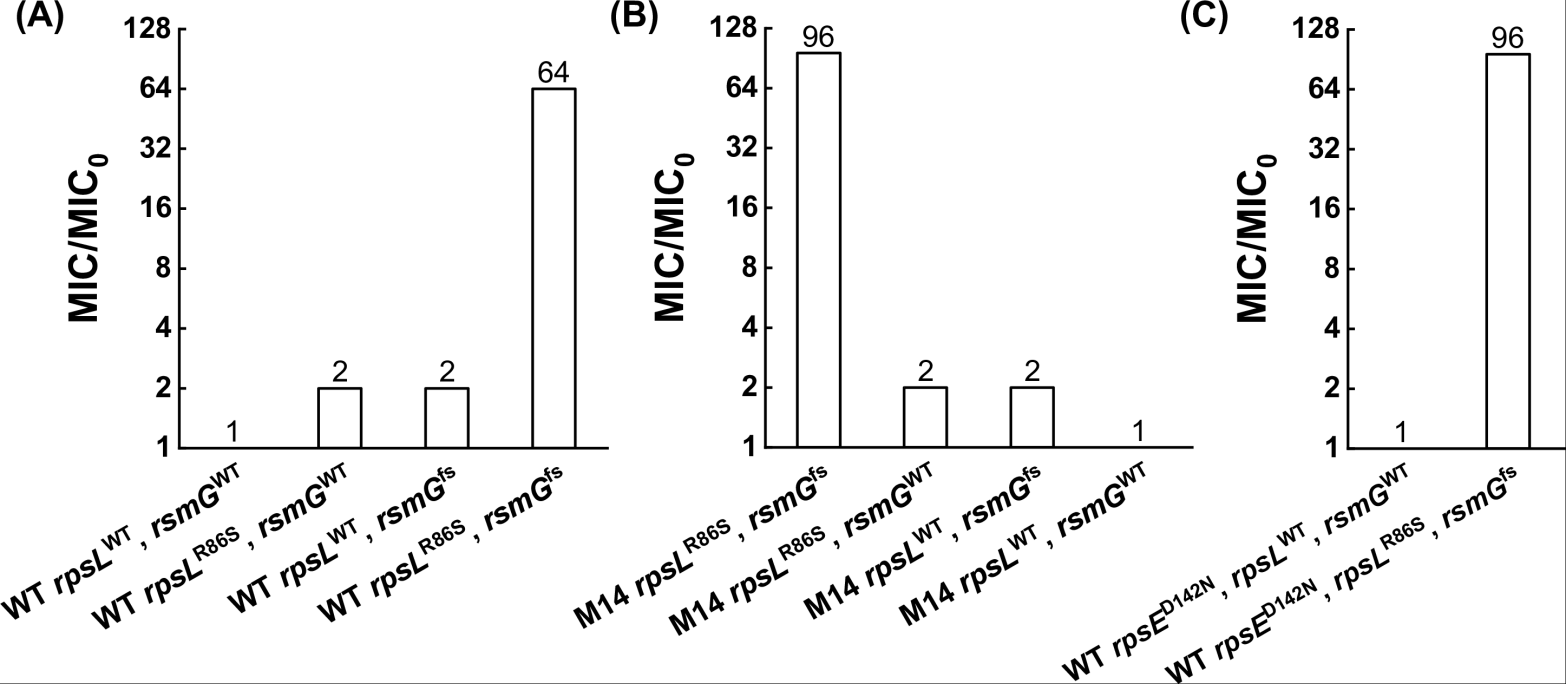
Effects of *rpsL*, *rsmG*, and *rpsE* mutations on streptomycin resistance. MIC fold changes relative to the wild type (WT, MIC_0_ = 32 mg/L) in different genetic backgrounds: (**A**) WT with *rpsL^R86S^* and *rsmG^W150fs^* mutations; (**B**) M14 with *rpsL^R86S^* and *rsmG^W150fs^*; and (**C**) WT with *rpsE^D142N^* combined with *rpsL^R86S^* and *rsmG^W150fs^*. Exact MIC/MIC_0_ fold change values are shown above bars. Three biological replicates were included for the MIC test, and the MIC was identical among replicates for each strain.

Our previous study identified two ribosomal mutations (i.e., *rpsL^R86S^* and *rsmG^W150fs^*) co-occurring in *E. coli* mutants that evolved high-level streptomycin resistance (>40-fold increase in MIC) under environmentally relevant selection pressures (i.e., 1/5 MIC of streptomycin and a mixture of pesticides at environmental levels) (13, 14). To disentangle the contribution of individual and combined mutations, we reconstructed each genotype in the wild-type, streptomycin-susceptible *E. coli* strain. Individual *rpsL^R86S^* or *rsmG^W150fs^* only led to a twofold increase in MIC (Fig. 1A). In contrast, their combination resulted in a 64-fold increase—resulting in an epistasis coefficient of 60—thereby demonstrating a strong positive epistasis between two mutations (Fig. 1A). To further validate this effect, we reverted the mutations to their wild-type alleles in the native resistant isolate M14, which evolved from the same wild-type strain under environmentally relevant exposures. As expected, reverting either *rpsL^R86S^* or *rsmG^W150fs^* caused a drastic drop in resistance (from 96-fold to 2-fold MIC_0_), and reverting both mutations fully restored streptomycin susceptibility (Fig. 1B).

Notably, although introducing *rpsL^R86S^* and *rsmG^W150fs^* into the wild-type background reproduced the majority (67%) of the resistance observed in M14, the reconstructed strain still exhibited 33% lower resistance than that of M14 (64× vs 96× MIC_0_). There seemed to be other mutations contributing to the overall resistance phenotype of M14. We further sequenced the genome of M14 and analyzed all mutations. Among them, we found another ribosomal mutation *rpsE^D142N^*. While introducing *rpsE^D142N^* alone into the wild type did not alter resistance, combining it with *rpsL^R86S^* and *rsmG^W150fs^* led to an additional increase in resistance to the same level as that of M14 (Fig. 1C).

Together, we demonstrated that it was the positive epistasis among three ribosomal mutations, *rpsL^R86S^*, *rsmG^W150fs^*, and *rpsE^D142N^*, that conferred the high-level streptomycin resistance of M14 previously isolated from *E. coli* populations under environmentally relevant co-exposure to streptomycin and pesticides (13). The interaction between *rpsL^R86S^* and *rsmG^W150fs^* played a more significant role, with a small and incremental contribution by the third mutation, *rpsE^D142N^*. These findings indicate the critical role of epistasis among co-evolved mutations in modulating high-level antibiotic resistance phenotypes.

The observed positive epistasis among ribosomal mutations may arise from their combined effects on the structure and function of the decoding center. Streptomycin can bind to RpsL and RpsE (the S12 and S5 protein, respectively), rendering inhibition of the initiation of protein synthesis or production of erroneous proteins. The *rsmG* gene encodes a 16S rRNA methyltransferase that modifies the ribosomal RNA near the decoding site. Mutations to these genes may interactively reduce streptomycin binding and restore translational accuracy, resulting in enhanced resistance. In this context, future studies can compare the translational accuracy between the wild-type and mutant cells under sublethal streptomycin conditions using a translation fidelity reporter, such as luciferase-based reporter systems (26), to further clarify how these mutation combinations reshape translational fidelity.

### Potential interactions of *rsmG^W150fs^* with other *rpsL* mutations identified in streptomycin-resistant *E. coli* strains

In addition to *rpsL^R86S^*, several other *rpsL* point mutations have been reported in *E. coli* isolates exhibiting varying levels of streptomycin resistance (Table 1). However, the lack of whole-genome sequencing data of resistant *E. coli* strains obtained from random mutagenesis or antibiotic selection makes it unclear whether the resistance was solely caused by the reported *rpsL* mutation or by epistasis with co-evolved mutations—potentially including *rsmG^W150fs^*, as demonstrated above. To address this knowledge gap, we investigated the interactions between *rsmG^W150fs^* and eight previously reported *rpsL* point mutations (Table 1). Three *rpsL* point mutations (H77P, K43N, and K88R) were successfully introduced into the wild-type strain, whereas the other five (P42L, K44E, L74P, P91L, and G92D) could not be obtained in either the wild-type or the *rsmG^W150fs^* mutant strain. Because *rpsL* encodes the essential ribosomal protein S12 (27), these point mutations could be lethal or inhibitory to cell growth.

**TABLE 1:** *rpsL* point mutations investigated in this study

SNP^*a*^	Amino acid change	MIC (mg/L) of evolved resistant *E. coli* isolates^*b*^ reported in literature (reference)	MIC of *E. coli* mutant with the respective single *rpsL* mutation in this study^*c*^ (mg/L)
C125T	P42L	>1,200 (28)	Lethal mutation^*d*^
A128C	K43N	>10,000 (29)	>32,768
A130G	K44E	>1,200 (28)	Lethal mutation
T221C	L74P	25 (30)	Lethal mutation
A230C	H77P	25 (30)	64
C256A	R86S	100 (31); 25 (30)	64
A263G	K88R	>10,000 (29)	>32,768
C272T	P91L	>10,000 (29); >1,200 (28)	Lethal mutation
G275A	G92D	>10,000 (29); >1,200 (28)	Lethal mutation

^*a*^ SNP identified by Sanger sequencing of PCR products targeting *rpsL*.

^*b*^ *E. coli* isolates were obtained in the lab by random mutagenesis or exposure to high-level streptomycin, and in parentheses is the reference cited.

^*c*^ Streptomycin MIC for the wild-type *E. coli* strain used in this study was 32 mg/L.

^*d*^ Introduction of these *rpsL* point mutations into either the wild-type strain or the *rsmG^W150fs^* mutant was unsuccessful, likely due to lethality of those *rpsL* mutations, suggesting the presence of potential co-occurring unknown compensatory mutations.

To rule out technical artifacts, we requested a commercial lab (GenScript Biotech, https://www.genscript.com) to introduce the *rpsL^P42L^* mutation into the wild-type and the *rsmG^W150fs^* mutant using CRISPR-Cas9-based mutagenesis. Consistently, no viable mutants can be recovered. Thus, these observations suggest that certain *rpsL* mutations may impose strong constraints on cellular viability, with the underlying causes remaining unclear. In native isolates with those *rpsL* mutations, there should be compensatory mutations present that were not captured in previous studies but played an essential role in rescuing the cell viability. Due to the lack of genome information, little further investigation could be done. This implies a critical role potentially played by multi-locus interactions in conferring a sustainable antibiotic resistance phenotype. It also underscores the need to identify all those interacting mutations as combined and more accurate antibiotic resistance biomarkers.

Among the successfully reconstructed *rpsL* point mutations, *rpsL^K43N^* and *rpsL^K88R^* are the two most frequently detected mutations, and their contribution to streptomycin resistance has been previously reported (10). Consistent with previous findings, both single mutants exhibited extremely high MIC (>32,768 mg/L, >1,024× MIC_0_), with detectable cell growth even at the highest streptomycin concentration tested. Introducing the *rsmG^W150fs^* mutation did not further increase the MIC, likely because resistance conferred by the *rpsL* mutation alone already exceeded the highest level tested. Thus, these *rpsL* alleles can independently confer extreme streptomycin resistance without requiring additional epistatic supports.

**Fig 2:**
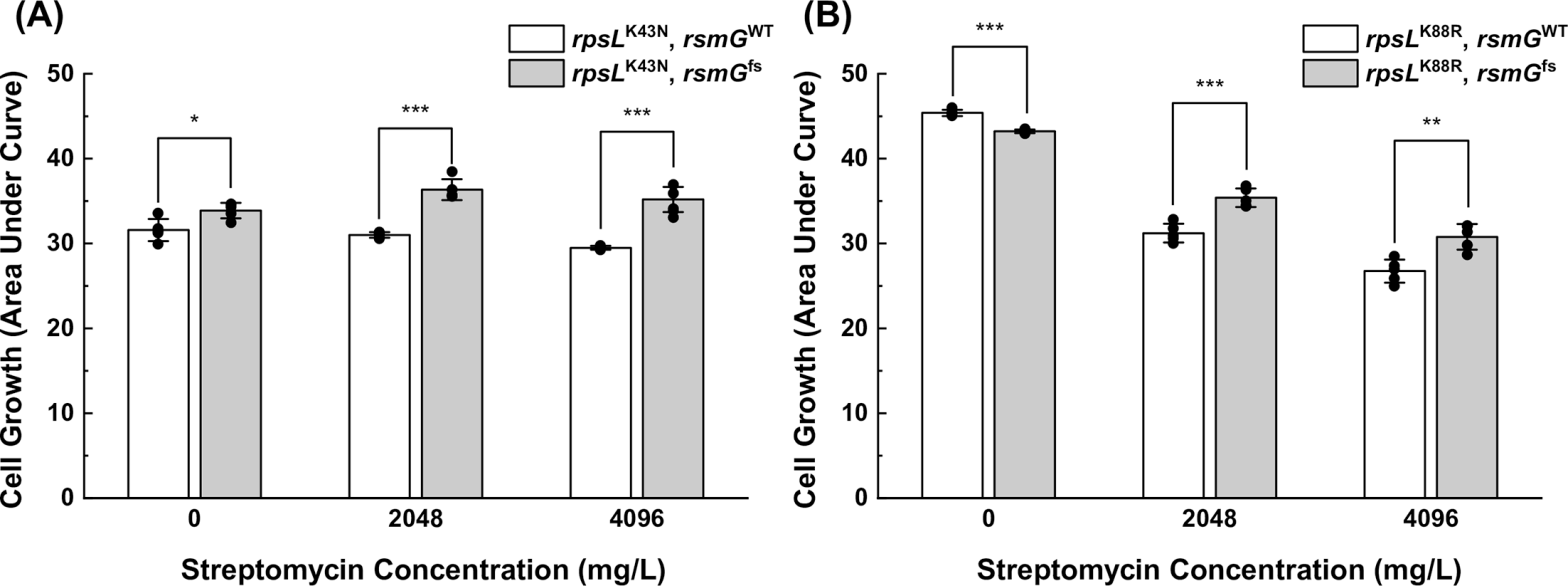
Growth of *rpsL* mutant strains with either *rsmG^WT^* or *rsmG^W150fs^* across increasing streptomycin concentrations. Cell growth was quantified as AUC. (**A**) *rpsL^K43N^* and (**B**) *rpsL^K88R^*. Data represent mean ± SD of five biological replicates indicated by each dot. Asterisks denote the statistical significance of Student’s *t*-test (*, *P* < 0.05; **, *P* < 0.01; ***, *P* < 0.001; *n* = 5).

Meanwhile, we took a closer look at the effect of the additional *rsmG^W150fs^* mutation on growth fitness under typical streptomycin stresses. Growth curves were obtained under different streptomycin concentrations, and the area under the curve (AUC) was used to assess population growth under antibiotic selective and non-selective conditions. Under streptomycin stress, the double mutants with both *rpsL* (K43N or K88R) and *rsmG^W150fs^* exhibited higher growth than the corresponding single *rpsL* mutant (Fig. 2 and Fig. S1). Notably, the additional *rsmG^W150fs^* mutation also promoted a better fitness of the *rpsL^K43N^* mutant in the absence of streptomycin (Fig. 2A). Therefore, although extremely high streptomycin resistance can be resulted by acquiring either of the two specific *rpsL* mutations, possessing another ribosomal mutation, *rsmG^W150fs^*, could provide a modest but measurable fitness advantage under both antibiotic selective and non-selective conditions.

**Fig 3:**
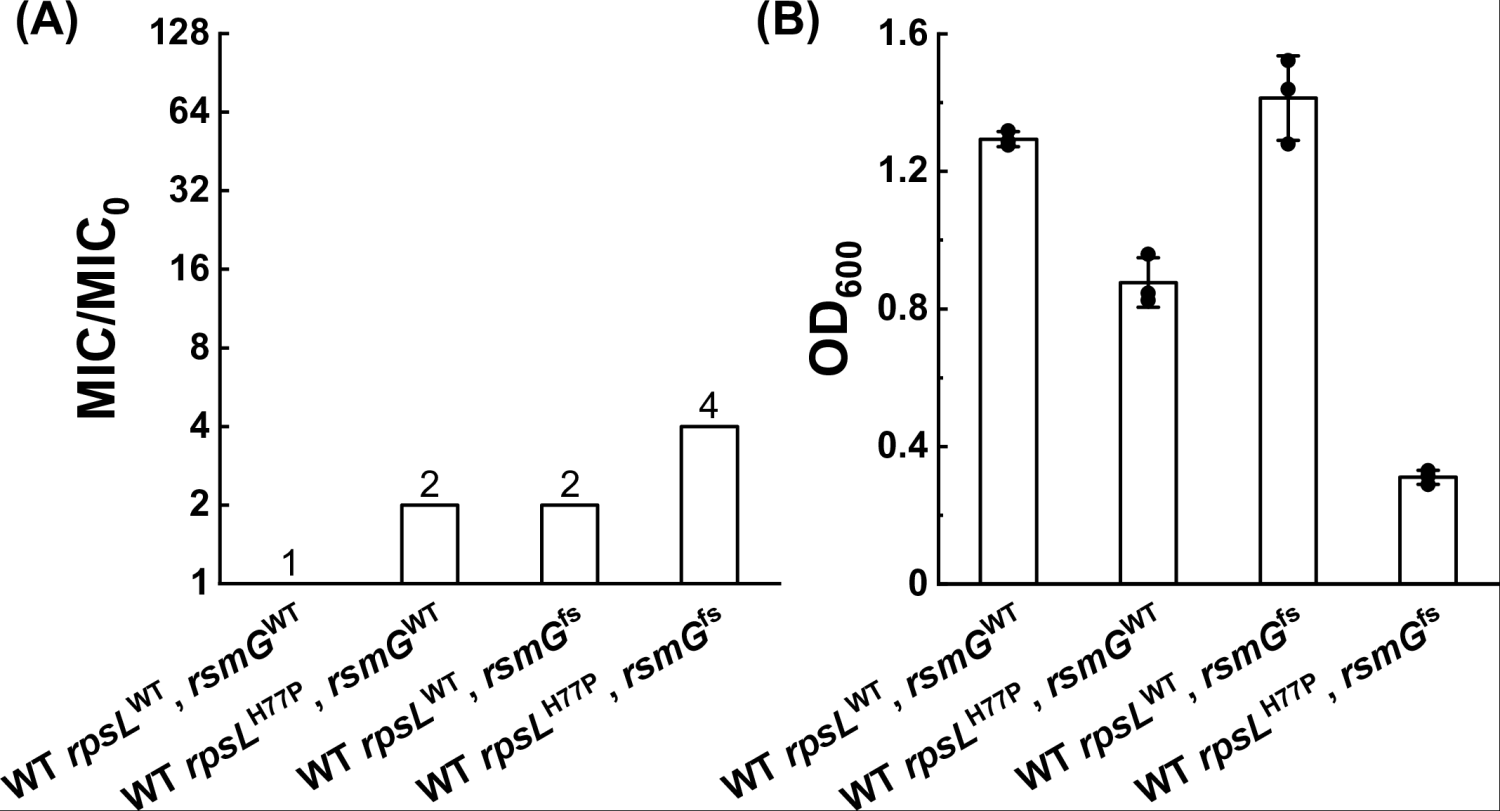
Effects of *rpsL*^*H77P*^ mutations on (**A**) streptomycin resistance and (**B**) cell growth with either *rsmG^WT^* or *rsmG^W150fs^* in streptomycin-free Luria–Bertani (LB) medium. MIC_0_ = 32 mg/L. Exact MIC/MIC_0_ fold change values are shown above bars. Three biological replicates were included for the MIC test, and the MIC was identical among replicates for each strain. Optical density at 600 nm (OD_600_) was measured after 20 h. Data represent mean ± SD of three biological replicates indicated by each dot.

Introducing *rpsL^H77P^* led to a mild increase in MIC (2×), similar to that observed in its native host (Table 1). When both *rpsL^H77P^* and *rsmG^W150fs^* were present, the MIC was increased by four times (Fig. 3A), suggesting an additive effect different from the epistasis observed between *rpsL^R86S^* (the single mutation conferred mild resistance) and *rsmG^W150fs^*. It is worth noting that the *rpsL^H77P^* single mutant and the *rpsL^H77P^ + rsmGW^W150fs^* double mutant exhibited a remarkable fitness cost with a more significant decrease in cell growth for the double mutant (Fig. 3B). The impaired cell growth could compromise the resistance phenotype of those mutants.

Collectively, other previously reported *rpsL* mutations did not show clear resistance epistasis with *rsmG*^*W150fs*^, although having *rsmG*^*W150fs*^ added some growth benefit for the *rpsL^K43N^* or *rpsL^K88R^* mutants. One possible reason is that resistance epistasis was specific to *rpsL* mutation sites. It can also be explained by the natural selection process, where only sustainable mutations could be selected and remain under the antibiotic selection pressure. Lethal mutations and mutations with substantial fitness cost may not sustain, and it was the case for several *rpsL* single mutants and the *rpsL^H77P^ + rsmG* double mutant in our study. Our previous studies revealed that *rsmG* loss-of-function mutations (e.g., *rsmG^W150fs^*) co-evolved with one specific *rpsL* site mutation, *rpsL^R86S^* (13, 14). In the same study (14), *rpsL^K88R^* and *rpsL^K43N^* mutations were detected without any accompanying *rsmG* mutations in *E. coli* populations under the co-exposure of streptomycin and pesticides. The *rpsL^K43N^* or *rpsL^K88R^* single mutation already confers extremely high resistance without significant growth cost. As a result, the additional growth benefit provided by the *rsmG* mutation is likely marginal and insufficient for the double mutant to be strongly selected or emerged during evolution. However, whether *rsmG* co-evolved with other *rpsL* mutations in Table 1 remains elusive due to unavailable genome information of the corresponding resistant mutants. It again emphasizes the need for whole-genome analysis of antibiotic-resistant bacteria to identify co-evolved mutations (e.g., *E. coli* strains with lethal *rpsL* single mutations) that could compensate for fitness cost and confer strong antibiotic resistance.

The findings of this study fill the knowledge gap and advance the fundamental understanding of antibiotic resistance mechanisms mediated by target-altering mutations, thereby benefiting antibiotic resistance surveillance. In addition to the horizontal transfer of antibiotic resistance genes, *de novo* mutations causing target alterations represent another major route of resistance development to many classes of antibiotics. Such mutations can reduce antibiotic binding affinity, thereby conferring resistance. For example, mutations of genes encoding proteins involved in protein and DNA synthesis have been identified in aminoglycoside and fluoroquinolone-resistant bacteria, respectively. In natural and agricultural settings, bacteria are frequently exposed to sub-inhibitory concentrations of antibiotics along with other chemical stressors such as pesticides (32). Antibiotic resistance can be selected at concentrations well below the MIC, often in the range of ~0.01–0.2× MIC, from µg/L to mg/L, depending on the antibiotic types and bacterial strains (13–18). The non-lethal exposure could facilitate more diverse *de novo* mutations across both antibiotic target and non-target genes. Here, we demonstrated that the high-level streptomycin resistance observed in resistant isolates under non-lethal exposures was attributed to positive epistasis among ribosomal mutations (e.g., *rpsL* and *rsmG*), each of which alone only conferred mild resistance. This suggests that the contribution of such resistance epistasis in resistance development in a broader environmental context could have been largely overlooked, particularly because most previous studies focused on individual genetic mutations. The *rpsL* mutation at the 43 and 88 amino acid positions and the loss-of-function *rsmG* mutation have also been identified in other environmental or clinical isolates (14, 33). The same epistatic *rpsL^R86S^* and *rsmG* mutations were detected in another *E. coli* strain (O103:H2) under the same lab exposure conditions, but not for the *Pseudomonas* and *Staphylococcus* strains in the same study (14). Thus, whether the positive epistasis among ribosomal mutations could be environmentally selected in other strains under the same selection conditions may depend on the strain’s genomic background.

Under environmentally relevant exposures, the same epistasis-mediated resistance mechanisms may be found among mutations of genes encoding a wide range of antibiotic targets, including DNA gyrases and topoisomerases (GyrAB and ParCE) for fluoroquinolones and RNA polymerases (e.g., RpoB) for rifampicin (19, 34). Therefore, to capture such interactions, future studies should employ whole-genome analysis rather than single-gene sequencing to get holistic mutation profiles of antibiotic-resistant mutants/populations, and even environmental microbial communities. This would allow us to comprehensively interrogate potential epistasis among co-evolved mutations for an extended range of antibiotics. Whole-genome/population sequencing has been achieved for large sample sizes at an affordable cost. The advancement of long-length and deep sequencing and the decreasing sequencing cost could help realize the detection of genetic mutations in complex environmental samples. Thus, validated individual and epistasis-based resistance mutations can be incorporated with resistome analyses to curate sequencing-based surveillance and promote a more accurate antibiotic resistance monitoring and risk assessment.

## MATERIALS AND METHODS

### Cell growth and bacterial strains

*E. coli* K-12 ATCC 10798 was obtained from the American Type Culture Collection (Manassas, VA, USA). The streptomycin-resistant strain M14 was isolated from an *E. coli* K-12 ATCC 10798 population exposed to sub-MIC streptomycin with mixed pesticides after 500 generations (13). All other strains were generated by genetic manipulation of these two strains, as described below. Cells were grown in LB broth (10 g tryptone, 5 g yeast extract, and 10 g NaCl per liter).

### Genetic manipulation

The λ-red homologous recombination system was employed for the genetic manipulation of *E. coli* (35). The *rsmG* frameshift mutation (W150fs) was first introduced without leaving a selection marker. However, the markerless gene replacement workflow will not work for essential genes like *rpsE* and *rpsL*. Alternatively, a kanamycin resistance cassette (*Kan^R^*) was inserted downstream of *rpsL* and a chloramphenicol resistance cassette (*Cm^R^*) downstream of *rpsE*, without gene deletion. Retention of these selection markers in the genome did not affect streptomycin resistance (Table S1). All constructed strains, as well as primers and plasmids used, are listed in Tables S2 through S4. A comprehensive list was compiled consisting of *rpsL* site mutations in streptomycin-resistant *E. coli* strains reported in the literature (Table 1). The corresponding site-specific mutants were constructed to examine their potential epistasis with the other commonly found ribosomal mutation, *rsmG^W150fs^*. For constructed mutant strains, the introduced mutation was verified by Sanger sequencing to confirm the intended genetic changes.

### Growth curve measurement

Growth curves were measured in clear 96-well microplates. Archived wild-type and genetically manipulated mutants were revived and streaked on LB agar plates with respective selection markers (for mutants). A single colony was first picked up and inoculated into LB broth. After growing overnight at 37°C and 180 rpm, the cell culture was diluted to an OD_600_ of 0.1 with 1× phosphate buffer saline (PBS). Subsequently, 0.5 µL of the standardized cell suspension was added to 199.5 µL of LB medium. The 96-well microplate was placed in the BioTek Synergy H1 microplate reader (Agilent Technologies, Santa Clara, CA, USA) at 37°C with orbital shaking, and OD_600_ values were measured every 15 min for 24 h. Five biological replicates were included. AUC was calculated using the following equation and used to represent the total cell growth:


$${AUC}_{total}=\sum {AUC}_{segments}=\sum_{i=1}^{n-1}\frac{{OD}_{i}+{OD}_{i+1}}{2}\times \left(t_{i+1}-t_{i}\right)$$

### MIC test

MIC was determined as the lowest antibiotic concentration that inhibited ≥90% of cell growth relative to the no-antibiotic control, based on OD_600_ measurements from 96-well microplate growth assays (36). A single colony was first picked up and inoculated into LB broth. After growing overnight at 37°C and 180 rpm, the cell culture was diluted to an OD_600_ of 0.1 with 1× PBS. Subsequently, 0.5 µL of the standardized cell suspension was added to 199.5 µL of LB medium supplemented with serial concentrations of streptomycin from 32 mg/L (MIC_0_) up to 32,768 mg/L (64× MIC_0_). The 96-well microplate was then incubated at 37°C for 20 h. After incubation, OD_600_ values were recorded by the BioTek Synergy H1 microplate reader. Three biological replicates were included for the MIC test, and the MIC was identical among replicates for each strain.

### DNA extraction, whole-genome sequencing, and variant calling

Genomic DNA of the streptomycin-resistant strain M14 was extracted using DNeasy Blood and Tissue Kits (Qiagen, Hilden, Germany), following the manufacturer’s instructions. The gDNA concentration was determined with a Qubit 4 Fluorometer (Thermo Fisher Scientific, Wilmington, DE, USA). Whole-genome sequencing was performed by SeqCenter (https://seqcenter.com, Pittsburgh, PA, USA) using an Illumina NovaSeq X Plus sequencer (2 × 150 bp). Raw data were trimmed by Trimmomatic v0.39 (37), with a minimum length of 36 bp. Trimmed data were aligned against the genome of *E. coli* K-12 ATCC no. 10798 downloaded from the ATCC website using Bowtie2 v2.5.1 (38). SAMtools v1.2.2 and Picard v3.4.0 were applied to index and mark duplicates (39). Variant calling was performed by BCFtools v1.2.2 and SnpEff (39, 40), with default parameters. All identified mutations were manually checked in the genome, and genes of interest were confirmed by Sanger sequencing using the primers listed in Table S1.

## Data Availability

The sequenced genome has been deposited in the NCBI Sequence Read Archive with the accession number PRJNA1332319.

## SUPPLEMENTAL MATERIAL

Supplemental material aem.00377-26-s0001.pdf Tables S1 to S4; Fig. S1.

ASM does not own the copyrights to Supplemental Material that may be linked to, or accessed through, an article. The authors have granted ASM a non-exclusive, world-wide license to publish the Supplemental Material files. Please contact the corresponding author directly for reuse.
