# Supplementary material for: Positive epistasis among ribosomal mutations drives high-level streptomycin resistance in Escherichia coli
Source: Appl Environ Microbiol. 2026 Jun 18;92(7):e00377-26. doi: 10.1128/aem.00377-26 (PMC13390409; doi:10.1128/aem.00377-26)
Supplement: Supplemental material — Tables S1 to S4; Fig. S1. [file aem.00377-26-s0001.pdf]

Supporting Information for

"Positive Epistasis among Ribosomal Mutations Drives High-Level Streptomycin Resistance in *Escherichia coli*"

running title: positive epistasis in antibiotic resistance

Fengjun Xu[1], Yue Xing[1], Yujie Men[1,✉]

[1]Department of Chemical and Environmental Engineering, University of California, Riverside, Riverside CA, 92521 USA

✉**Corresponding authors:**

Dr. Yujie Men

Email: ymen@engr.ucr.edu

Office: +1 (951) 827-1019

Address: Bourns Hall, 900 University Ave, Riverside, CA 92521, USA

Table S1. Influence of introducing *KanR/CmR* on streptomycin resistance.

#Strain	Description	MIC (mg/L)
FX-500	wild-type *E. coli* K-12 ATCC No.10798 strain, rpsL(WT)::rsmG(WT)	32
FX-526	wild-type strain, rpsL(WT)::KanR::rsmG(WT)	32
FX-553	wild-type strain, rpsE(WT)::CmR::rpsL(WT)::KanR::rsmG(WT)	32

Table S2. Primers used in this study.

Primer Name	Primer Seuqence (5' - 3')
rpsL_42_F	CTTAAAAAACCGAACTCCGCGC
rpsL_42_R	GCGCGGAGTTCGGTTTTTTAAG
rpsL_43_F	CCTACAAAACCGAACTCCGCGC
rpsL_43_R	GCGCGGAGTTCGGTTTTGTAGG
rpsL_44_F	CCTAAAGAACCGAACTCCGCGC
rpsL_44_R	GCGCGGAGTTCGGTTCTTTAGG
rpsL_74_F	GTGGTGAAGGTCACAACCCGCA
rpsL_74_R	TGCGGGTTGTGACCTTCACCAC
rpsL_77_F	AGGAGCCCTCCGTGATCCTGA
rpsL_77_R	TCAGGATCACGGAGGGCTCCT
rpsL_88_F	CGTGTTAGAGACCTCCCGGGT
rpsL_88_R	ACCCGGGAGGTCTCTAACACG
rpsL_91_F	CGTGTTAAAGACCTCCTGGGT
rpsL_91_R	ACCCAGGAGGTCTTTAACACG
rpsL_92_F	CGTGTTAAAGACCTCCCGGAT
rpsL_92_R	ATCCGGGAGGTCTTTAACACG
rpsL_F	ATGGCAACAGTTAACCAGCTGG
rpsL_R	AATCCATCTTGTTCAATCATGCG<br>TTAAGCCTTAGGACGCTTCACGC
in_rpsL_KanR_F	GCGTGAAGCGTCCTAAGGCTTAACGC<br>ATGATTGAACAAGATGGATT
in_rpsL_KanR_R	TTAGTTTGACATTTAAGTTAAAACGTTTGGCCTTACTTAACGGAGAACCA<br>TCAGAAGAACTCGTCAAG
rsmG_F	GTGCTCAACAAACTCTCCTTACTGC

rsmG_R	AATCCATCTTGTTCAATCATGCG<br>TTAAATTTTATTTGCTTTAATCACCACCAG
rsmG_KanR_F	GTGGTGATTAAAGCAAATAAAATTTAA<br>TTGTGTAGGCTGGAGCTGCTTC
rsmG_KanR_R	TGTTGTTAACAGTCTAACCGGTCAATTTTTTATGATTTTTTTGATAAAAA<br>CATATGAATATCCTCCTTAGTTCCTATTCC
rpsE_F	ATGGCTCACATCGAAAAACAAGCTG
rpsE_R	CTAAGGAGGATATTCATATG<br>TTATTTCCCCAGAATTTCTTCAACGGATTT
in_rpsE_CmR_F	CATATGAATATCCTCCTTAGTTCCTATTCC
in_rpsE_CmR_R	CGACCGATTGCACTGCGGGTTTGAGTAATTTTAATAGTCTTTGCCATGGT<br>TTGTGTAGGCTGGAGCTGCTTC

Table S3. Plasmids used in this study.

Plasmid Name	Source
pKD3	Addgene #45604 (1)
pKD4	Addgene #45605 (1)
pKM208	Addgene #13077 (2)
pBAD-Flp	Addgene #122969 (3)

Table S4. Bacterial strains used in this study.

#Strain	Description	Reference
FX-500	wild-type *E. coli* K-12 ATCC No.10798 strain	ATCC
FX-501	isolate M14	(4)
FX-518	wild-type strain, rpsL(86)::KanR::rsmG(WT)	This study
FX-520	wild-type strain, △rsmG::rsmG(fs)	This study
FX-526	wild-type strain, rpsL(WT)::KanR::rsmG(WT)	This study
FX-527	wild-type strain, rpsL(WT)::KanR::rsmG(fs)	This study
FX-528	wild-type strain, rpsL(86)::KanR::rsmG(fs)	This study
FX-529	isolate M14, rpsL(WT)::KanR::rsmG(WT)	This study
FX-530	isolate M14, rpsL(86)::KanR::rsmG(WT)	This study
FX-531	isolate M14, rpsL(WT)::KanR::rsmG(fs)	This study
FX-532	isolate M14, rpsL(86)::KanR::rsmG(fs)	This study
FX-533	wild-type strain, rpsL(43)::KanR::rsmG(WT)	This study
FX-534	wild-type strain, rpsL(43)::KanR::rsmG(fs)	This study
FX-538	wild-type strain, rpsL(88)::KanR::rsmG(WT)	This study
FX-539	wild-type strain, rpsL(88)::KanR::rsmG(fs)	This study
FX-544	wild-type strain, rpsL(77)::KanR::rsmG(WT)	This study
FX-545	wild-type strain, rpsL(77)::KanR::rsmG(fs)	This study
FX-553	wild-type strain, rpsE(WT)::CmR::rpsL(WT)::KanR::rsmG(WT)	This study
FX-554	wild-type strain, rpsE(142)::CmR::rpsL(WT)::KanR::rsmG(WT)	This study
FX-555	wild-type strain, rpsE(WT)::CmR::rpsL(86)::KanR::rsmG(fs)	This study
FX-556	wild-type strain, rpsE(142)::CmR::rpsL(86)::KanR::rsmG(fs)	This study

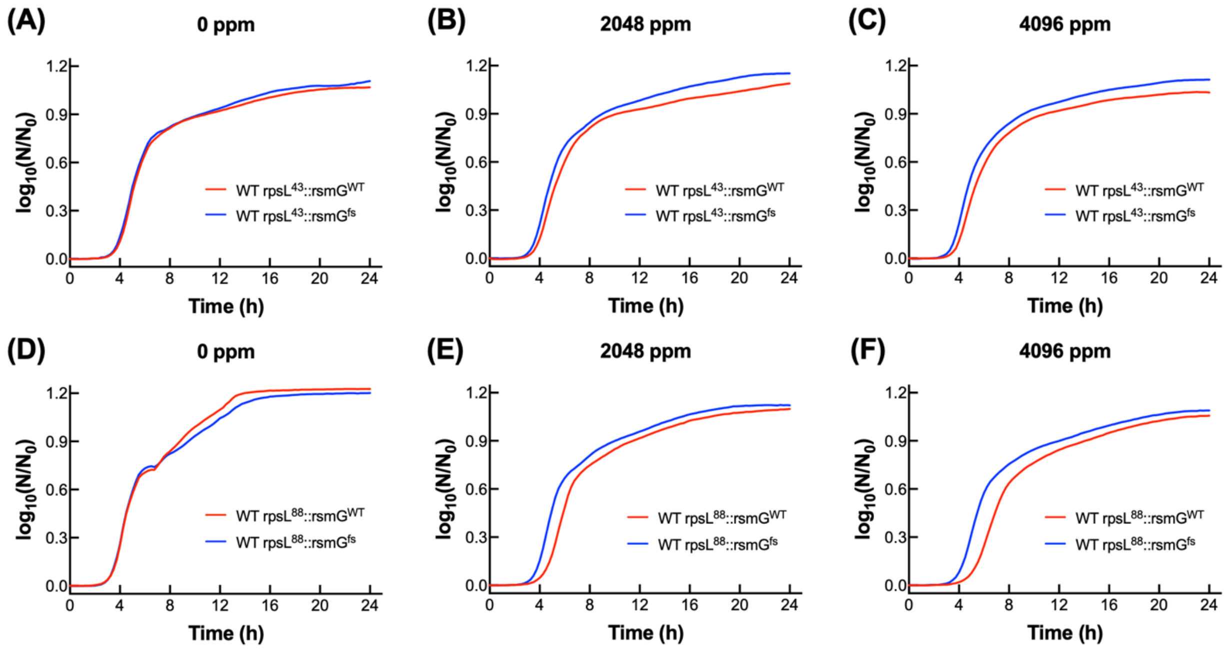

Figure S1. Growth curve of *rpsL* mutant strains with either *rsmG*^{WT} or *rsmG*^{W150fs} across increasing streptomycin concentrations. OD$_{600}$ (optical density at 600 nm) was measured every 15 min and log transformed. (A-C) *rpsL*^{K43N}; (D-F) *rpsL*^{K88R}. Three biological replicates were included for each group.

References

1. Datsenko KA, Wanner BL. 2000. One-step inactivation of chromosomal genes in *Escherichia coli* K-12 using PCR products. *Proc Natl Acad Sci U S A* 97:6640-5.
2. Murphy KC, Campellone KG. 2003. Lambda Red-mediated recombinogenic engineering of enterohemorrhagic and enteropathogenic *E. coli*. *BMC Mol Biol* 4:11.
3. Dalia TN, Chlebek JL, Dalia AB. 2020. A modular chromosomally integrated toolkit for ectopic gene expression in *Vibrio cholerae*. *Sci Rep* 10:15398.
4. Xing Y, Kang X, Zhang S, Men Y. 2021. Specific phenotypic, genomic, and fitness evolutionary trajectories toward streptomycin resistance induced by pesticide co-stressors in *Escherichia coli*. *ISME Commun* 1:39.
